# Comorbidities and Mortality in Hypercapnic Obese under Domiciliary Noninvasive Ventilation

**DOI:** 10.1371/journal.pone.0052006

**Published:** 2013-01-16

**Authors:** Jean-Christian Borel, Benoit Burel, Renaud Tamisier, Sonia Dias-Domingos, Jean-Philippe Baguet, Patrick Levy, Jean-Louis Pepin

**Affiliations:** 1 INSERM U 1042, HP2 Laboratory, Université Joseph Fourier, Faculté de Médecine, Grenoble, France; 2 CHU, Hôpital A. Michallon, Pôle Locomotion, Rééducation et Physiologie, Grenoble, France; 3 AGIRadom, Research and Development department, Meylan, France; 4 CHU, Hôpital A. Michallon, Département de cardiologie, Grenoble, France; Thomas Jefferson University, United States of America

## Abstract

**Background:**

The higher mortality rate in untreated patients with obesity-associated hypoventilation is a strong rationale for long-term noninvasive ventilation (NIV). The impacts of comorbidities, medications and NIV compliance on survival of these patients remain largely unexplored.

**Methods:**

Observational cohort of hypercapnic obese patients initiated on NIV between March 2003 and July 2008. Survival curves were estimated by the Kaplan–Meier method. Anthropometric measurements, pulmonary function, blood gases, nocturnal SpO_2_ indices, comorbidities, medications, conditions of NIV initiation and NIV compliance were used as covariates. Univariate and multivariate Cox models allowed to assess predictive factors of mortality.

**Results:**

One hundred and seven patients (56% women), in whom NIV was initiated in acute (36%) or chronic conditions, were followed during 43±14 months. The 1, 2, 3 years survival rates were 99%, 94%, and 89%, respectively. In univariate analysis, death was associated with older age (>61 years), low FEV1 (<66% predicted value), male gender, BMI×time, concomitant COPD, NIV initiation in acute condition, use of inhaled corticosteroids, ß-blockers, nonthiazide diuretics, angiotensin-converting enzyme inhibitors and combination of cardiovascular drugs (one diuretic and at least one other cardiovascular agent). In multivariate analysis, combination of cardiovascular agents was the only factor independently associated with higher risk of death (HR = 5.3; 95% CI 1.18; 23.9). Female gender was associated with lower risk of death.

**Conclusion:**

Cardiovascular comorbidities represent the main factor predicting mortality in patient with obesity-associated hypoventilation treated by NIV. In this population, NIV should be associated with a combination of treatment modalities to reduce cardiovascular risk.

## Introduction

Obesity is a chronic condition associated with metabolic, hormonal, cardiovascular and respiratory impairments causing an increase in death rate [1]. Obstructive sleep apnea syndrome (OSAS), commonly associated with obesity [2], is also a risk factor for cardio-metabolic morbidity [3], [4]. Beyond OSAS, a subgroup of obese patients is affected by chronic respiratory failure, characterized by diurnal hypercapnia [5]. Two main syndromes can be encountered in obesity-associated chronic hypercapnia. Firstly, the obesity hypoventilation syndrome (OHS), defined as a combination of obesity (BMI ≥30 kg/m^2^), daytime hypercapnia (PaCO_2_≥45 mmHg) and various types of sleep-disordered breathing after ruling out other disorders that may cause alveolar hypoventilation [6]. Secondly, the overlap syndrome defined as the combination of OSAS and chronic obstructive pulmonary disease (COPD) [7], [8], [9]. Indeed, COPD, OSAS and obesity acted synergistically to increase the risk of sleep hypoxemia and hypercapnia [9], and therefore causes chronic respiratory failure [7], [10]. Both OHS and overlap syndrome are characterized by a high rate of cardiovascular morbimortality [5], [11], [12].

Noninvasive ventilation (NIV) effectively improves some characteristics of obesity-associated hypoventilation [13]. Particularly, sleep-related breathing disorders encountered in obese patients are a modifiable obesity-related cardio-vascular risk factor. In observational cohorts [14], [15], [16] NIV seems to be effective in reducing mortality in obese patients suffering from sleep breathing disorders but the overall mortality rate remains higher than long-term mortality rates observed in large cohorts of obese patients submitted to bariatric surgery [17]. It is possible that some key factors predicting mortality in obese patients treated with NIV have not been yet identified. The main objective of this study was to assess the factors related to risk of death in a cohort of patients with obesity-associated hypoventilation treated with home-based long term NIV. We included a full description of comorbidities and medications as covariates of mortality, which have never been included in previous mortality studies.

## Materials and Methods

### Study Design and Patients’ Selection

Observational cohort study of patients with obesity (BMI ≥30 kg.m^−2^) and hypercapnia (PaCO_2_≥45 mmHg) treated after hospital discharge with at-home, long-term, noninvasive ventilation (NIV). Patients who started NIV between March 2003 and July 2008 were identified through a regional home-care database. NIV was initiated during hospitalization for acute or chronic hypoventilation in five different medical facilities (one tertiary university hospital, one general hospital and three private practice centers). The standard definition for inclusion was “patients in whom obesity was the main explanation for hypoventilation”. Patients suffering from neuromuscular disorders, untreated hypothyroidism, progressive restrictive parenchymal lung diseases (such as fibrosis) were not included. The presence of any other respiratory disease (history of COPD [when 30%<FEV1/VC <70%], asthma, moderate thoracic restrictive diseases) that could participate to hypoventilation was not an exclusion criterion.

Ethical committee approval was obtained by “Le Comité consultatif sur le traitement de l’information en matière de recherche en santé” (C.C.T.I.R.S 11.371) and authorization from the “Commission Nationale Informatique et Liberté” (C.N.I.L), the French information technology and personal data protection authority. According to the C.N.I.L and C.C.T.I.R.S’s recommendations for this retrospective study, an information letter was mailed to each patient but a written consent was not required. The information letter mentioned that the patients could refuse that their information to be used for the study. Regarding the patients who had died, an exemption for the requirement of information was obtained by the CNIL.

### Data Collection

Patients’ medical charts were retrospectively analyzed in each of the five centers by the same investigator (B.B.). Medical and smoking history, anthropometric data, daytime and sleep respiratory parameters and usual medical treatment before NIV initiation were extracted. The condition of NIV initiation (acute respiratory failure or chronic respiratory failure) was also reported. Baseline reference arterial blood gases (ABG) should have been drawn maximum one day before NIV initiation. In acute hypercapnic respiratory failure condition, ABG values were considered as acceptable even though they were performed under supplemental O_2_. Pulmonary function tests must have been performed within a month preceding NIV initiation in order to be considered as valid data.

### Follow-up and Outcomes

After hospital discharge, patients were systematically followed every three months by the same home care provider until death or NIV discontinuation. Health authorities, in France, require visits by home care nurses or technicians before providing reimbursement for the ventilatory support. Vital status was ascertained by home care provider or in case of NIV discontinuation by patient’s general practitioner. The analysis was censored on October 1, 2010. Adherence to NIV was assessed via the time counter of the ventilator. NIV adherence was considered at 0 hour/day in patients who discontinued NIV during the survey. During follow up, patients who were switched to continuous positive airway pressure therapy (CPAP) were considered and analyzed as pursuing NIV.

### Data Management and Statistical Analysis

Data were analyzed using Statistical Analysis System (SAS®) software version 9.1.3 (SAS Institute, Cary, NC, USA). Continuous data were expressed as mean ± standard deviation (SD), and categorical data as percentage. Baseline variables were compared according to conditions of NIV initiation using Kruskal-Wallis rank sum test or ANOVA.

Univariate Cox proportional hazards regression models were used to determine baseline parameters (i.e. at NIV initiation) that were associated with survival. Continuous data were converted to dichotomous data (> or<to the median value). Since BMI did not respect proportional risks hypothesis (time-dependent variable), it was converted to a new variable: BMI x time. To describe the frequency and outcomes according to baseline variables, all- cause mortality were constructed according to the Kaplan-Meier analysis.

Variables associated with survival in univariate analysis with p<0.2 were included in a multivariable Cox proportional hazards regression model (backward selection). Co-linearity between variables (defined as p<0.2 and r>0.4) was assessed by Pearson’s or Spearman’s coefficient or Cramer’s V2. Variables which had more than 15% of missing values were discarded. For other variables, the missing values were replaced by the variable’s median for continuous data and for categorical data by the most frequent value. Only a p-value <0.05 was considered statistically significant.

## Results

### Flow Chart

**Figure 1:**
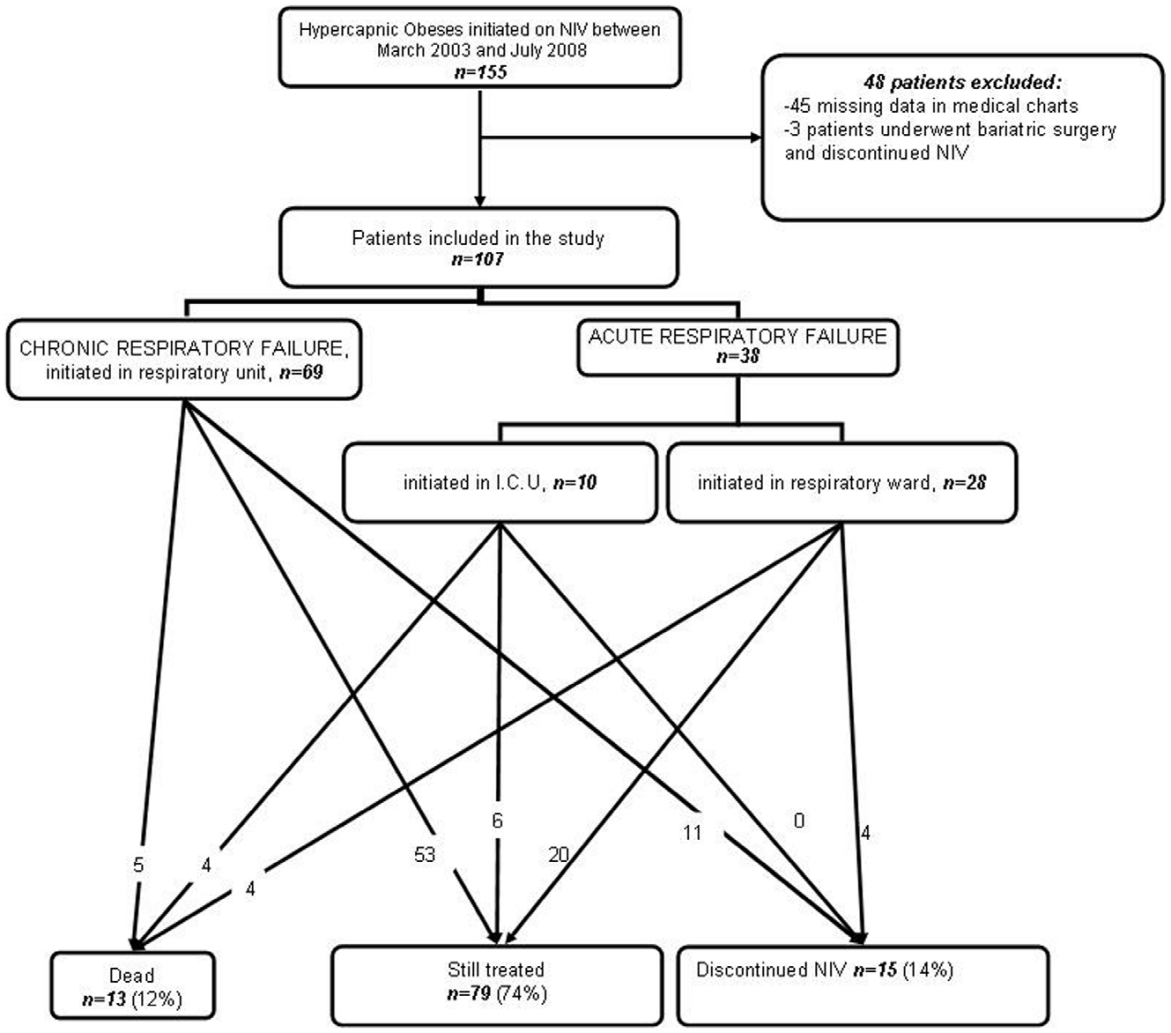
Flow chart of the survey. CRF: Chronic Respiratory Failure; AHRF: Acute Hypercapnic Respiratory Failure; I.C.U: Intensive Care Unit.

As depicted in Figure 1, a total of 107 patients who started NIV between March 2003 and July 2008 were included in the study. NIV was initiated in either acute hypercapnic respiratory failure (AHRF) (26.4% in respiratory ward or 9.3% in intensive care unit [ICU], respectively) or in a chronic stable state of hypercapnic respiratory failure (64.4%).

### Baseline Assessment

Table 1 shows the baseline characteristics according to patient’s NIV initiation conditions. As expected, PaCO_2_, pH and respiratory function were more severely affected in patients referred for acute respiratory failure. Medical history revealed high rates of cardiovascular comorbidities regardless of NIV initiation conditions. However, patients referred in acute respiratory failure were significantly more likely to have a previous diagnosis of heart failure. Accordingly, these patients were more frequently treated with cardiovascular medications (antiplatelets agents, anticoagulants, non-thiazidic diuretics). Moreover, AHRF patients exhibited a higher rate of COPD and were more often former smokers. Before the inclusion in the study, seven patients (6,5%) had been previously treated by CPAP but were no longer treated at the time of inclusion; sixteen patients (15%) had been treated by CPAP until the inclusion and were then switched to NIV owing to persistent hypercapnia.

**Table 1:** Baseline Characteristics according to NIV initiation conditions.

	Chronic/respiratory ward, n = 69		Acute/respiratory ward, n = 28	Acute/I.C.U, n = 10	p-value	
Age, *years*	59±13		63±10	65±8	ns	
Gender, *% male*	42		39	70	ns	
BMI, *kg/m^2^*	40.2±6.7		41.4±7.3	37.9±5.5	ns	
PaO_2_, *kPa (*n of ABG with O_2_)	9.7±1.5 (0)		8.9±2.4 (10)	9.1±2.7 (7)	ns	
PaCO_2_, *kPa*	6.1±0.7*#		6.7±1.3 #	8.5±2.3*	<0.001	
pH	7.40±0.03 *		7.39±0.04	7.32±0.07 *	0.001	
VC, *% predicted value*	76±21 *		65±20*	62±22	0.03	
FEV_1_/VC, *%*	77±14		81±14	70±16	ns	
Apnea-Hyponea index, *n/h (*n of missing data)	42±32 (9)		44±32 (11)	37±36 (6)	ns	
Mean nocturnal SpO2, *% h (*n of missing data)	91±4 (10)		89±7 (5)	89±4 (6)	ns	
SpO2<90%, *%recording time h (*n of missing data)	27±30 (12)		37±31 (8)	44±29 (6)	ns	
Medical History						
Former smoker, *%*		46.4	50.0*	90.0*	0.04	
Hypertension, *%*		81.2	82.1	80.0	ns	
Heart Failure,%		34.8*	60.7*	70.0	0.02	
coronary heart disease, %		14.5	10.7	40.0	<0.1	
Pulmonary Hypertension,%		8.7 #	28.6 #	30.0	0.02	
Thrombo-embolism, %		5.8	14.3	10.0	ns	
Stroke, %		5.8	14.3	10.0	ns	
Type 2 diabetes, %		34.8	39.3	40.0	ns	
Dyslipidemia, %		42.0	39.3	40.0	ns	
COPD, %		21.7	35.7	60.0	0.03	
Asthma, %		13.0	7.1	30.0	ns	
Sleep apnea, %		72.5	57.1	60.0	ns	
Depression, %		29.0	32.1	40.0	ns	
Medications						
ß-blokers, %		29.0	28.6	20.0	ns	
Diuretics, %		52.2	60.7	80.0	ns	
Non-thiazide diuretics, %		24.6*#	57.1*	80.0#	<0.001	
Calcium antagonists, %		17.4	39.3	30.0	<0.1	
CEI, %		24.6	28.6	50.0	ns	
ARBs, %		34.8%	25.0	30.0	ns	
Anti-platelet drugs, %		18.8#	35.7	60.0#	0.01	
Anticoagulants, %		4.3*	25.0*	20.0	0.01	
Statins, %		34.8	42.9	50.0	ns	
Insulin, %		11.6	3.6	20.0	ns	
Inhaled corticoids, %		27.5	21.4	60.0	<0.1	
Oral antidiabetics, %		31.9	39.3	40.0	ns	
Psychoacticve drugs, %		71.0	57.1	70.0	ns	
NIV settings						
Inspiratory pressure* *(cmH_2_O)*		18.9±3.0	19.4±3.7	20.8±5.0	ns	
Expiratory pressure *(cmH_2_O)*		9.6±1.9	10.0±2.2	9.1±2.6	ns	
Back-up frequency *(/min)*		12.7±2.6	13.0±3.6	14.1±1.7	ns	

Chronic = Chronic Respiratory Failure; Acute: Acute Respiratory Failure; I.C.U: Intensive Care Unit; BMI: Body Mass Index; n of ABG with O2: number of arterial blood gases realized with additional O_2;_ VC: Vital Capacity expressed as percentage of predicted value; FEV1/VC: Forced Expiratory Volume in one second/Vital Capacity ratio; Heart failure included (ischemic, hypertrophic or dilated); COPD: Chronic Obstructive Pulmonary Disease; OSAS: Obstructive Sleep Apnea Syndrome; CEI: Angiotensin Converting Enzyme Inhhnibitors; ARBs: Angiotensin II Receptor Blockers.

* #: represent significant difference between pairwise comparisons.

### Follow-up (Figure 1 and Table 2)

**Table 2:** Follow-up data according to the condition of NIV initiation.

	Chronic/respiratory ward, n = 69	Acute/respiratory ward, n = 28	Acute/I.C.U, n = 10	p-value
Mean daily use of NIV, *h/day*	5.2±3.4	5.3±3.9	5.7±3.3	ns
Mean follow-up duration, *months*	45±13	41±15	39±1	ns
Rate of death, *%*	7.2*	14.3	40.0*	0.01
Additonal long term O_2_, *%*	13.0 #*	32.1 *	60.0 #	0.001

For the group as a whole, the mean observation time was 43±14 months. Thirteen patients (12%) died after a mean duration of 25±12 months. The 1, 2, 3 years survival probabilities were 99%, 94% and 89%, respectively. Fourteen patients (14%) with persistent non adherence discontinued NIV. Seven patients were switched to CPAP therapy. The mean daily use for NIV was 5.4±3.5 hours/day. The mean follow-up duration as well as the mean NIV adherence was similar whatever NIV initiation conditions. Nevertheless, patients with AHRF exhibited higher rates of death and they were more likely to receive additional long term oxygen therapy to NIV.

### Factors Associated with Risk of All-cause Mortality

**Figure 2:**
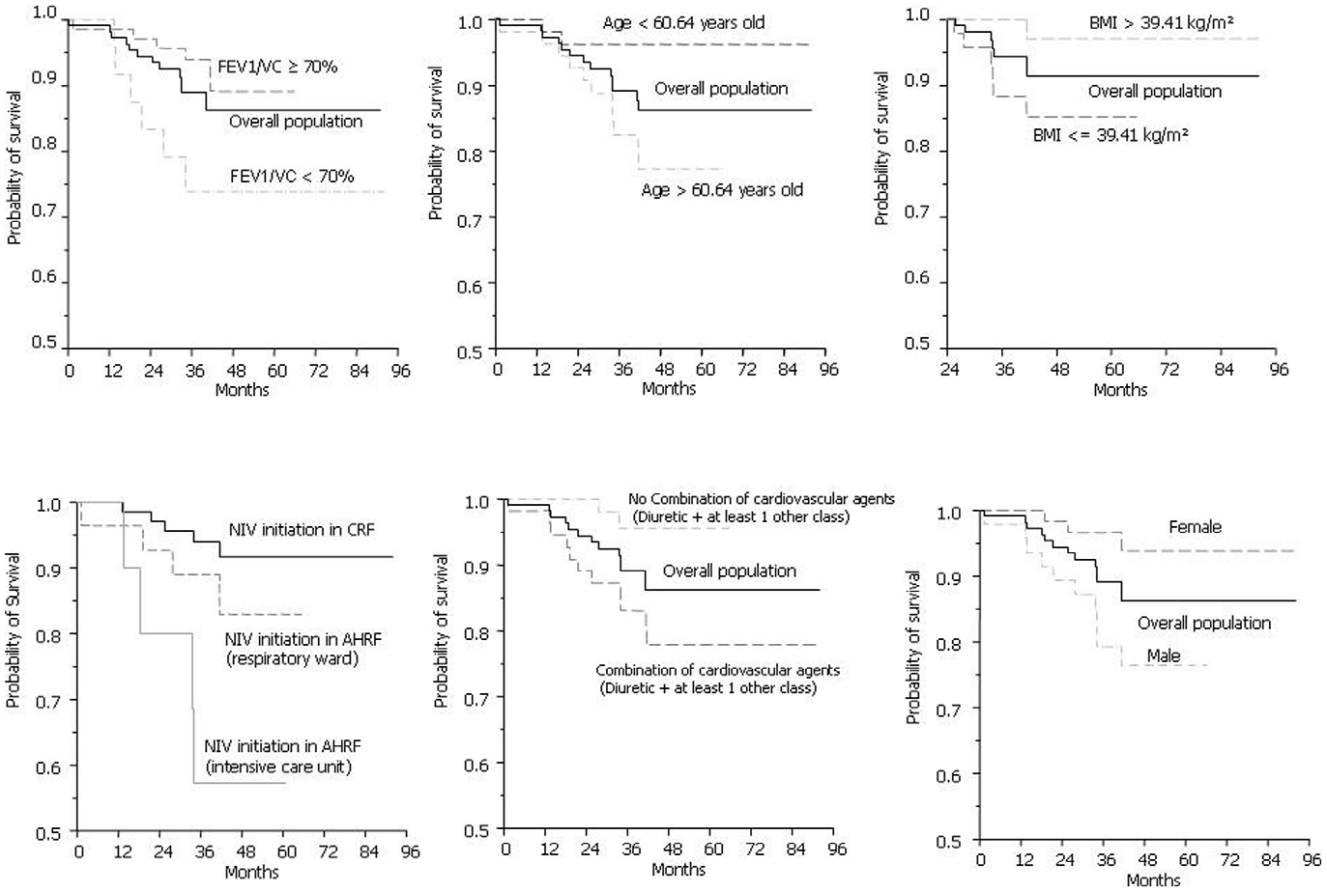
Kaplan-Meier survival curves referring to BMI, gender, FEV1/VC, use of combination of cardiovascular agents and condition of NIV initiation.

**Figure 3:**
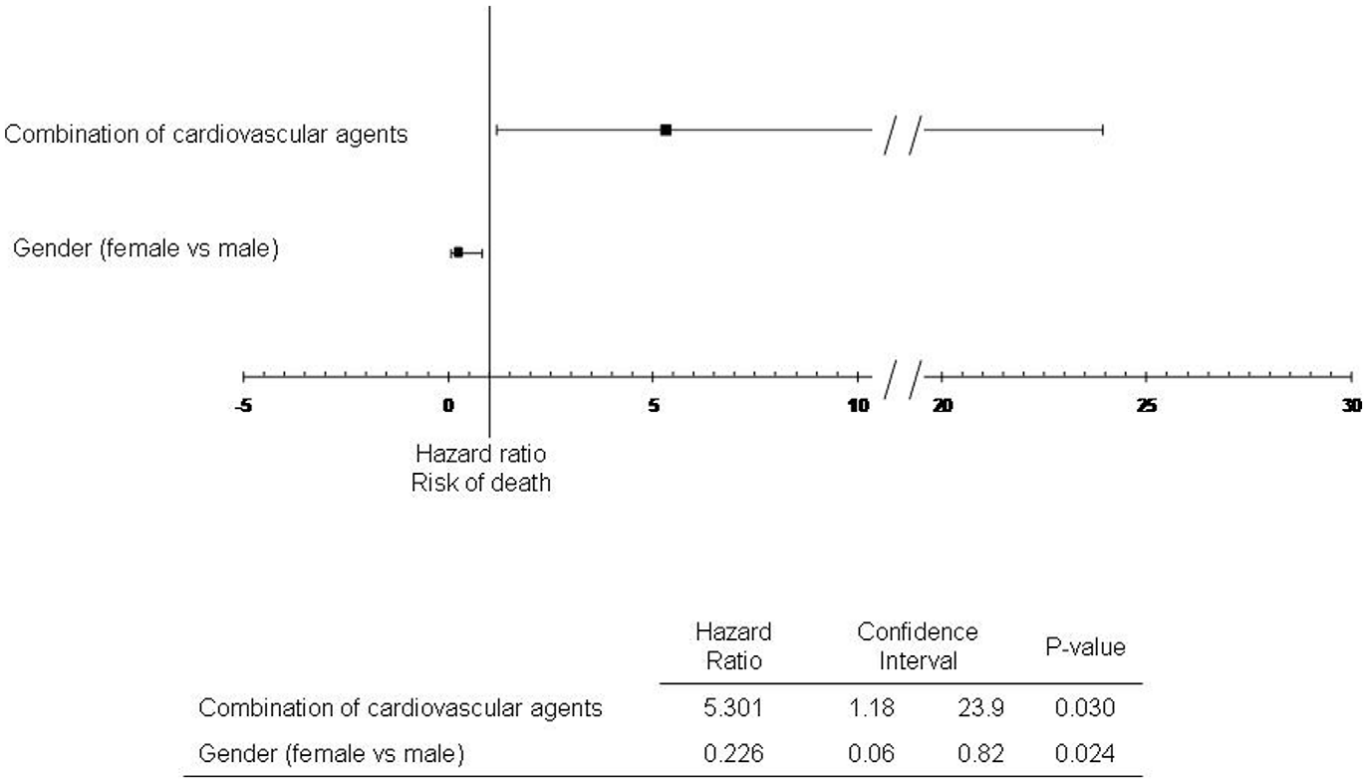
Independent factors associated with risk of all-cause mortality (multivariate Cox model). Combination of cardiovascular agents: Combination of a diuretic and at least one other cardiovascular agent among ß-Blokers, Calcium antagonists, Converting Enzime Inhibitors, Antagiotensin II receptor blockers.

Figure 2 shows the Kaplan-Meier survival curves of factors which were significantly associated with risk of all-cause mortality in univariate analysis. Table 3 displays baseline characteristics associated with the risk of death with a p-value <0.2 in univariate analysis. The choice between collinear variables was based on clinical considerations. The following were included in a multivariate Cox proportional hazards regression model: Age, gender, BMI×time, NIV initiation conditions, vital capacity, FEV1/VC (reflecting COPD history and intake of inhaled corticosteroids), Combination of cardiovascular agents, history of diabetes, former smoker status, additional long-term O_2_, psychoactive drugs. Combination of cardiovascular agents was the only factor independently associated with higher risk of death in multivariate analysis. Female gender was associated with lower risk of death (Figure 3).

**Table 3:** Factors associated with risk of all-cause mortality (univariate Cox model).

	Hazard ratio	95% confidence interval	p-value
Age (>60.6 years)	5.464	1.2; 24.6	0.03
Gender (female vs male)	0.221	0.06; 0.8	0.02
Time *BMI (≥39.4 kg.m^−2^ after 24 months)	0.079	0.01; 0.6	0.01
NIV initiation (Acute versus Chronic)	3.24	1.1; 9.9	0.04
VC (>72% predicted value)	0.228	0.05; 1.0	<0.1
FEV_1_ (>66% of predicted value)	0.2	0.04; 0.9	0.04
FEV_1_/VC (>77.5%)	0.332	0.09; 1.2	<0.1
AIH (>33/h)	3.098	0.6; 15.3	<0.2
Mean nocturnal SpO_2_ (>91.4%)	0.264	0.06; 1.2	<0.1
**Medical History**			
Former smoker	3.226	0.9; 11.7	<0.1
Type 2 Diabetes	2.224	0.7; 6.6	<0.2
COPD	3.112	1.0; 9.7	0.05
Dyslipidemia	1.218	0.4; 3.6	>0.7
Stroke	1.964	0.4; 8.9	0.3801
Sleep apnea	1.648	0.4; 6.0	>0.4
Hypertension	–	–	>0.9
Heart Failure	2.36	0.8; 7.0	<0.2
coronary heart disease	1.47	0.4; 5.4	>0.5
Pulmonary Hypertension	1.986	0.5; 7.2	>0.2
Thrombo-embolism	2.166	0.5; 9.8	>0.3
Asthma	1.269	0.3; 5.7	>0.7
Depression	0.686	0.2; 2.5	>0.5
**Usual Medical treatment**			
ß-blokers, %	3.418	1.1; 10.2	0.03
Diuretics, %	4.274	0.9; 19.3	<0.1
Non-thiazide diuretics, %	6.257	1.7; 22.8	0.005
CEI, %	3.022	1.0; 9.0	0.05
Combination of cardiovascular agents, %	5.429	1.02; 24.5	0.03
Oral antidiabetics, %	2.568	0.9; 7.7	<0.1
Psychoacticve drugs, %	0.368	0.1; 1.7	<0.2
Inhaled corticoids	4.507	1.5; 13.8	0.01
Calcium antagonists	0.936	0.26; 3.4	>0.9
ARBs	0.599	0.17; 2.2	>0.4
Anti-platelet drugs	1.639	0.54; 5.0	>0.3
Anticoagulants	0.682	0.09; 5.2	>0.7
Statins	0.947	0.3; 2.9	>0.9
Insulin	1.86	0.4; 8.4	>0.4
**Follow-up**			
Additonal long term O_2_	2.208	0.7; 6.8	<0.2

Time*BMI: Body Mass Index; VC: Vital Capacity expressed as percentage of predicted value; FEV1: Forced Expiratory Volume in one second; AIH: Apnea Hypopnea index expressed as number of events per hour of sleep; Combination of cardiovascular agents: Combination of a diuretic and at least one other cardiovascular agent among ß-Blokers, Calcium antagonists, Converting Enzime Inhibitors, Antagiotensin II receptor blockers; COPD: Chronic Obstructive Pulmonary Disease. Continuous data were converted to dichotomous data (> or<to the median value); Since BMI did not respect proportional risks hypothesis (time-dependant variable), it was converted to a new variable: BMI x time. This variable should be interpreted as follows: After 24 months, BMI above 39.4 kg m^−2^ is associated with lower risk of death than BMI bellow 39.4 kg.m^−2.^

## Discussion

The main objective of this study was to assess the factors associated with risk of death in a cohort of obese hypercapnic patients treated with home-based long-term NIV. The results can be summarized as follows: (i) A third of obese hypercapnic patients with the most severe comorbidities were diagnosed on the occasion of an acute respiratory failure and were then initiated on NIV in the ICU or in respiratory ward. (ii) Beyond the condition of NIV initiation, gender, age, BMI, history of COPD, and cardiovascular comorbidities (recognized by the use of a combination of cardiovascular medications) were associated with higher risk of death when treated by NIV. (iii) In multivariate analysis, cardiovascular comorbidities remained the only factor independently associated with a higher risk of death.

### Obesity-associated Hypoventilation Remains a Largely Undiagnosed Syndrome

The finding that NIV was initiated in acute respiratory failure in a third of the patients of the present cohort is in accordance with previous studies [18], [19]. However, these studies were including patients initiated on NIV in the 1990s. Our study underscores that there is always –within current knowledge- a lack of screening for hypoventilation in obese patients and that initiation of NIV is dramatically delayed [20]. Moreover, patients initiated under NIV in acute conditions were more likely to have previously developed cardiovascular complications favoring multiple organ failure and a higher rate of death. When initiated early, there is growing evidence to suggest that NIV would be efficient in reducing requirement of intensive care, general health care resources [21]as well as improving survival [14], [15]. Taken together these results reinforce the need for better screening for hypercapnia in patients with obesity-associated hypoventilation, particularly in primary care resources. In obese patients, serum bicarbonate level >27 mMol/L is associated with a high prevalence of OHS. This marker would be sensitive - low cost – and easily available to better screen obesity-related hypoventilation and thus anticipate patient’s referral to respiratory physicians [22].

### Survival Rates in Patients Treated by NIV for Obesity-associated Hypoventilation

The two and three years mortality rates were comparable in the present study with those of previous published reports [14], [19]. Priou et al [19] and Budweiser et al [14] reported survival rates of 93 and 92% at two years of follow-up compared to 94% in the present study. However, in their studies, Budweiser et al [14] and Priou et al [19] applied tighter inclusion criteria for COPD patients and excluded subjects with FEV1/VC<50% and FEV1/IC <60%, respectively. In the present study, exclusion criteria were less restrictive (FEV1>30% of predicted value) to better document the association between obesity-associated hypoventilation and COPD in terms of mortality. Indeed, the association between untreated sleep breathing disorders and COPD is known to increase the risk of death. This has been demonstrated for patients with mild to moderate COPD (average BMI = 30.5±5.1 kg.m^−2^) [12] as well as for GOLD IV hypercapnic COPD (mean BMI = 32.3±5.4 kg.m^−2^) already established on long-term oxygen therapy [8]. Marin et al.^12^ showed that CPAP improved survival in patients with a mild to moderate overlap syndrome. In the Marin’s study, significant limitation was that PaCO_2_ values were not available and only a limited number of patients were treated by NIV.

Overlap syndrome and obesity hypoventilation syndrome are linked by sleep apnea, episodes of REM hypoventilation and a high frequency of cardiovascular comorbidities. We firmly believe that a clear cut distinction between these two syndromes is arbitrary and does not reflect the reality of clinical practice. Accordingly, Nowbar et al [5] in their paper on obesity-associated hypoventilation reported that 38% of patients had previously received a diagnosis of COPD. In the field of COPD, Garcia-Aymerich et al [23] described the cluster of *“systemic COPD”* characterized by obesity, a mild respiratory impairment and a high prevalence of cardiovascular diseases.

### Cardiovascular Comorbidities: The Best Predictor of Mortality in Obese Patients Treated with Noninvasive Ventilation

Our study extends knowledge by demonstrating that cardiovascular comorbidities are the major determinants of death in patients with obesity-associated hypoventilation treated by NIV. The use of a combination of cardiovascular agents as a marker of cardiovascular comorbidities probably underestimates the prevalence of comorbidities as the use of secondary prevention medications remains low [24]. However, even with a lack of sensitivity, the use of two or more cardiovascular agents represented a consistent surrogate marker of cardiovascular comorbidities. Indeed, in a recent study, Weber et al. [25] compared patients at heightened cardiovascular risk that are using a monotherapy versus a combination therapy. The two groups of patients had similar histories of stroke or coronary events and the comparison between the two groups was adjusted for prior cardiovascular disease and left ventricular hypertrophy (other major clinical characteristics, including the prevalence of diabetes mellitus, were not different in these two groups). These authors demonstrated a clear increase in mortality rate and a higher proportion of cardiovascular events in patients with a combination therapy. The hypothesis was that a combination of cardiovascular medications reflected a more severe history of previous cardiovascular disease. Another study [26] raised the same conclusions. Accordingly, we strongly believe that a combination of cardiovascular agents is a reliable marker of risk in our hypercapnic obese patients. There is an important and simple clinical message behind this: Hypercapnic obese patients treated at home with long term NIV and requiring combination of cardiovascular agents are at higher risk of mortality and justify a closer follow-up.

We have recently demonstrated in a randomized control trial that one-month NIV clearly improved diurnal and nocturnal respiratory outcomes in patients suffering from OHS, but cardiovascular parameters such as blood pressure, endothelial dysfunction as well as arterial stiffness remained unchanged [27]. Interestingly, low-grade inflammation was not influenced by NIV probably because in these morbidly obese patients, obesity per-se is the major explanation for persistent chronic inflammation. In severe COPD, chronic inflammation probably also persist even after treatment of sleep disordered breathing. Although NIV has been shown to be effective in reducing mortality in patients with obesity-associated hypoventilation, the long-term mortality rate for these patients remains higher when compared to data observed in large cohorts of obese patients who underwent bariatric surgery which treats obesity-related complications more entirely than NIV [17].

### Methodological Considerations

We acknowledge that we could not provide evidence of long term NIV effectiveness such as follow-up arterial blood gases, nocturnal oxymetry, polygraphy and clinical outcomes (dyspnea severity score, sleepiness, quality of life). Future prospective studies should specifically address the question oflong term variations in NIV efficacy and mortality.

### Conclusion and Perspectives

This study shows that better screening for patients with obesity-associated hypoventilation is necessary in order to reduce the delay of NIV initiation and avoid acute respiratory failure. Cardiovascular comorbidities remain the major determinant of death in these patients who are treated by NIV for sleep breathing abnormalities. Thus, NIV should be combined with other modalities of treatment such as rehabilitation programs including physical training, weight loss, lifestyle changes and appropriate medication to further improve cardiovascular risk factors in patients with obesity-associated hypoventilation.
